# Identification of motives and barriers to physical activity of polish young mothers

**DOI:** 10.1186/s12905-020-01061-y

**Published:** 2020-09-11

**Authors:** Andrzej Soroka, Agnieszka Godlewska, Elżbieta Krzęcio-Nieczyporuk, Paulina Kozioł

**Affiliations:** Siedlce University of Natural Sciences and Humanities, Faculty of Medical and Health Sciences, B. Prusa 14 st., 08-110 Siedlce, Poland

**Keywords:** Physical activity, Young mothers, Rural areas, Urban areas, Motives and barriers

## Abstract

**Background:**

The purpose of the study was to determine the level of physical activity of young mothers living in rural and urban areas and their free time budget. The article also aims to indicate motivating factors and barriers encountered during physical activity.

**Methods:**

A diagnostic survey method, including two survey IPAQ questionnaires – the long version and author’s questionnaire, has been applied in the study. A representative sample consisted of 1064 young mothers who gave birth in 2017. Student T test and discriminate function analysis have been used in statistical analysis.

**Results:**

No significant difference appeared between young mothers from rural areas and urban areas in the level of physical activity. At the same time, the authors of the study identified several smaller differences when analyzing particular aspects of physical activity. Young mothers from rural areas more often declared their participation in physical activity to improve their physical condition and reduce pain complaints. On the other hand, young mothers from urban areas emphasized that physical activity helps them to strengthen self-confidence and improve their position in the family. In case of the main barriers to physical activity, both group of respondents pointed to the lack of money and interest in occasional events.

**Conclusions:**

The main conclusion is that more efforts are needed is to strengthen family support and material status of young families. It is also important to eliminate infrastructure barriers, mostly in rural areas and raise awareness on the importance of health education.

## Background

Lifestyle is one of the four main determinants of human health with its most important element that is physical activity. Other important determinants are physical and social environment, genetic factors and organization of medical care. The physical activity is essential for the proper functioning of human body and health [1, 2]. It also helps to improve human well-being and plays an important role in the treatment of depression and anxiety [3, 4]. Modern public health studies investigate determinants of physical activity as they may help to fight the pandemic development of non–communicable diseases [5] such as cardiovascular disease, cancer, depression, hypertension, diabetes, and osteoporosis [6–8].

Current trends show that insufficient physical activity has become a global problem, steadily affecting more and more adults and children [9–13] The studies report that people with high socioeconomic status, including profession, income and educational level, very often spent free time on physical activity [14, 15]. The researchers also point that women are less active than men. This is especially evident in the case of women who lack sport and recreational skills, and have lower socioeconomic and material status [16]. What is more, women’s participation in physical activity during free time regularly declines throughout their life, especially during pregnancy [17, 18].

There is evidence to suggest that the onset of parenthood may cause a decline in physical activity of adults [19, 20]. This is often associated with anxiety, reduced mental well-being and the need to make lifestyle changes [21, 22]. Both mothers and fathers experience the above mentioned difficulties, however women to a greater extent than men [13].

According to social cognitive theory there is a relationship between environment and behavior [23]. There are studies which report the correlation between environmental factors and physical activity [24]. The physical activity behaviors of adults from rural areas depend on whether their neighbors are also physically active and whether they have access to exercise facilities, [25]. In general, the studies show that rural communities differ significantly from urban ones [26].

## Methods

### Study population

The quota sampling was used as a method for selecting survey participants. The total population of Polish women who gave births to live children in 2017 was 357,400 [27]. It was divided into two groups: women living in rural areas and those living in towns and cities. While determining the size of representative sample, which eventually consisted of 1064 respondents, the confidence level was set at 0.95, the estimated size fraction at 0.42, and the maximum error at 0.03. The survey was conducted on the basis of respondents’ availability, until the limit was reached for each province. Such approach did not create a selection bias. The study was carried out in April 2018 among young mothers who had given birth in 2017. In order to collect study material, the following web portals were used: forum.e-mama.pl, webmama.pl, familie.pl and maluchy.pl.

The study conforms to the code of ethics of the World Medical Association and the standards for research recommendations of the Helsinki Declaration. The protocol was approved by the local university ethics committee at the Siedlce University of Natural Sciences and Humanities. The written consent to participate in the study was obtained from all respondents. All respondents were over 18 years old. To ensure confidentiality, all data were anonymized before analysis. The survey was conducted in the form of telephone inquiries and the data collection procedure was applied. The standard interview with the respondents lasted 10 min.

### Data collection and definitions

A diagnostic survey with two research tools (survey questionnaires) were used in the study. The first tool was IPAQ (International Physical Activity Questionnaire) - long version, developed for measuring four areas of physical activity: profession, housework and outside the home, sports and recreation and everyday movement [28]. This questionnaire is widely used method for determining the level of physical activity [29]. The second questionnaire was the author’s survey questionnaire which was used to define leisure time budget of young mothers (Appendix No. 1). It was also applied to determine the hierarchy of motivating factors and barriers that respondents encounter while making attempt to participate in physical activity. The above-mentioned aims refer to a socio-demographic factor that is the place of residence that is rural and urban areas. A five-point Likert scale was used to measure attitudes. The use of questionnaire survey was preceded by two-stage pilot studies which allowed evaluating the content of the questionnaire, examining its reliability, validity, and discriminate power.

The goal of the study is to determine the level of physical activity of young mothers living in rural and urban areas and their free time budget. The study also aims to define the hierarchy of motivating factors and barriers that they encounter during physical activity.

The research hypothesis assumes that young mothers from rural areas have lower level of physical activity and less free time than mothers living in urban areas. Additionally, the main barriers that affect a level of participation in physical activity of young mothers from rural areas are the lack of time, limited financial resources, difficult access to sports and recreation infrastructure, and small support from their families.

### Statistical analysis

The *Statistica* 13.1 PL software was used in statistical analysis to calculate means and standard deviations. The authors also applied Shapiro-Wilk normality test in order to compare variations, Student t test to compare the mean values of variables, Chi-square compliance test and discriminant function analysis. They were used to determine which factors of studied phenomena discriminate two naturally emerging groups. The discriminant function analysis was used to calculate the classification function coefficients. Prior to discriminant analysis, the multivariate normality was verified by checking if each variable had a normal distribution. It was assumed that the variance-covariance matrix was homogeneous within groups. Slight deviations that appeared were not valid. It was due to the size of groups which were respectively: 618 in urban and 446 in rural areas. On the other hand, statistically significant were differences of mean values where the probability of uncertainty was less than *p* < 0.05.

## Results

The analytical material consisted of 1064 questionnaires (446 collected from rural areas, 618 from urban areas of Poland). The mean age of respondents living in rural areas was 25.3 ± 1.8, while those from urban areas 29.3 ± 4.9. The body mass of respondents from rural areas was 60.7 ± 8.9 kg and the body height 165.7 ± 3.5 cm. In case of young mothers from urban areas, the body mass was 62.7 ± 9.6 kg and the body height 167.3 ± 5.8 cm. The BMI calculation showed that 30.7% of young mothers from rural areas were underweight, 46.3% represented normal weight-growth ratio, while 22.9% were overweight. In case of respondents from urban areas, 25.6% were underweight, 59.7% had normal parameters, while 14.6% were overweight. The rest of analysed parameters are presented in Table 1.

**Table 1:** Participants’ data

Participants’ data		Place of residence	
		Urban areas	Rural areas
	Age	29,3 ± 4,9	25,3 ± 1,8
	Body mass	62,7 ± 9,6 kg	60,7 ± 8,9 kg
	Body heigh	167,3 ± 5,8 cm	165,7 ± 3,5 cm
BMI	Underweight	25,6%	30,7%
	Normal parametrs	59,8%	46,4%
	Overweight	14,6%	22,9%
Education	University degree	79,5%	53,9%
	Average education	17,8%	42,2%
	Professional or basic education	2,7%	3,9%
Economic status	High	8,2%	0%
	Average	50,1%	57,4%
	Low	41,7%	42,6%

The analysis of obtained results showed no significant differences in the level of physical activity between young mothers living in rural and urban areas. At the same time, the authors reported several smaller differences that appeared in particular areas of physical activity. The respondents from rural areas showed higher level of physical activity *p* < 0.001 in the area of professional work, movement and participation in recreation and sport activities. On the other hand, respondents from urban areas showed significantly (*p* < 0.001) higher level of physical activity related to housework (Table 2).

**Table 2:** The level of physical activity in different areas of Polish young mothers living in rural and urban areas (in MET-min./week)

Area of physical activity	Urban area		Rural area		*t*-test value	*p* value
	$\overline{x}$*±SD*	n	$\overline{x}$*±SD*	n		
Total activity	4691.3 ± 2562.3	618	4674.6 ± 2025.2	446	0.240	0.810
Professional work	682.5 ± 642.1	618	1050.6 ± 623.2	446	4.931	0.001*
Movement	612.5 ± 554.3	618	993.6 ± 523.2	446	4.603	0.001*
Housework	2548.6 ± 1765.2	618	1386.3 ± 723.8	446	4.101	0.001*
Sport and recreation activities	847.7 ± 423.9	618	1244.7 ± 762.2	446	6.402	0.001*

* - difference significant *p* < 0.005

The extremely high value of  test *p <* 0.001 shows that there is a strong dependence between the amount of free time and place of residence. Women from rural areas more often than women from urban areas declared their free time budget as more than 5 h a day, both weekdays and weekends. On the other hand, nearly half of respondents from both surveyed groups declared the limit below 2 h of free time a day (Table 3).

**Table 3:** Free time budget of Polish young mothers on weekdays and weekends with regard to their residence place

Days	Place of residence	Free time budget (%)			
		Below 2 h	From 2 to 5 h	Above 5 h	Total
Weekdays	Urban area	37.42	18.13	2.41	57.96
	Rural area	19.38	14.46	8.20	42.04
	Total	56.8	32.59	10.61	100.00
	Pearson chi-square		71.937	*p* = 0.001*	
Weekends	Urban area	26.62	19.38	11.96	57.96
	Rural area	19.38	0.00	22.66	42.04
	Total	46.00	19.38	34.62	100.00
	Pearson chi-square		226.598	*p =* 0.001*	

* - difference significant *p* < 0.05

What is more, the high value of  test indicated a significant difference between the observed values and expected ones. More respondents from rural areas than from urban ones reported that their free time budget was fully sufficient. On the other hand, the same group of respondents more often declared the need to have more free time. Interesting is the fact that there were no respondents from rural areas who suffered from a total lack of free time (Table 4).

**Table 4:** Dimension of leisure time of Polish young mothers living in rural and urban areas

Place of residence	Leisure time dimension (%)				
	It is enough	It could be more	It is not enough	No free time	Total
Urban area	6.36	13.40	26.42	11.76	57.97
Rural area	9.74	14.46	17.84	0.00	42.04
Total	16.10	27.87	44.26	11.76	100.00
Pearson chi-square		123.894		*p* = 0.001*	

* - difference significant *p* < 0.05

Among eleven motives that had influence on young mothers’ participation in physical activity, six of them were included in the model of discriminate function. Thus, the following motives were not included in this model: creating healthy lifestyle, improving beauty, improving personal well-being, strengthening immune system and weight loss. The study showed significantly different values of particular motives between two surveyed groups. The highest value of classification function was reached by a motive related to a desire to improve health condition through participation in physical activity,. This was significantly more important *p* < 0.001 for respondents from rural than from urban areas. On the other hand, such motives as strengthening self-confidence (*p* < 0.001), strengthening family position (*p <* 0.001) or reducing stress (*p* = 0.002) were significantly more important for women from urban areas than from urban areas (Table 5).

**Table 5:** Motives which encourage young mothers from urban and rural areas to spend their free time actively

Motive	Model of discriminant analysis			Classification functions	
	Wilks’ Lambda: 0.437 F (10.212) = 6.945 *p <* 0.001			Place of residence	
	Wilks’ Lambda	F of introduction	p level	Urban area *p* = 0.580	Rural area *p* = 0.420
Improving health condition	0.472	120.980	0.001*	1.498	2.803
Improving self-confidence	0.466	72.555	0.001*	1.632	0.611
Relieving the pain	0.513	41.147	0.001*	0.007	0.610
Strengthening family position	0.505	30.540	0.001*	1.139	0.761
Reducing stress	0.489	9.335	0.002*	0.177	0.101
Body hardening	0.484	2.445	0.118	0.123	0.068
Constant				7.761	9.591

* - difference significant *p* < 0.05

Source: Own data on a base of study results

The discriminate analysis and Wilks’ Lambda showed significant differences in the values of individual barriers in physical activity faced by young mothers from rural and urban areas. The study reported that the lack of financial resources was the most often declared barrier by all respondents. What is more, the lack of interest in sport and recreational offers was the second factor that reached high values in both groups of respondents (Table 6).

**Table 6:** Barriers that hinder young mothers living in rural and urban areas from spending their leisure time actively

Barrier	Model of discriminant analysis			Classification functions	
	Wilks’ Lambda: 0.507 F (10.212) = 6.945 *p <* 0.001			Place of residence	
	Wilks’ Lambda	F of introduction	p level	Urban area *p =* 0.580	Rural area *p =* 0.420
No offers of sport and recreational centers	0.508	9.273	0.002*	1.443	1.724
No family support	0.501	132.017	0.001*	0.408	1.328
The lack of free time	0.493	17.044	0.001*	0.928	1.318
No access to sport facilities	0.521	13.581	0.001*	1.726	1.386
No interests in offers	0.507	8.319	0.004*	5.252	5.003
No financial resources	0.486	6.919	0.008*	7.641	7.401
Constant				21.347	

* - difference significant *p <* 0.05

Source: Own data on a base of study results

## Discussion

There is no evidence for research hypothesis that assumed lower level of physical activity among young mothers from rural areas than those living in urban areas. The level of physical activity in both groups surveyed was similar in contrast to study results conducted in the United States [30, 31]. However, the young Polish mothers choose different types of physical activity. Female respondents from rural areas spent nearly half of their physical activity on housework and work on the farm. This is a common phenomenon which significantly increases daily levels of physical activity of this group of respondents [32].

Around 25% of total physical activity of both studied populations was related to sports and recreational activities. Dzewaltowski et al. [33] point out to a higher probability that children who are strongly attached to their families will take over the same parenting patterns in adulthood. This especially applies to female children who learn their parents’ approach to physical activity, mostly of mothers, and introduce these patterns to their own families in the future [34–37].

A part of research hypothesis concerning leisure time budget was not proven in the case of young mothers from rural areas. The study showed that young women from rural areas, especially on weekdays, had much more leisure time than women living in urban areas. It is worth mentioning that many women from urban areas function in multigenerational families. This helps them to save more free time as all daily duties are divided among a greater number of family members. However, in general the family and housework duties significantly limit women’s free time spent on physical activities [38]. It is worth mentioning that mothers of young children tend to meet the needs of their children in the first place, and then their own ones [39]. According to some authors [40, 41], this makes them unable to participate in physical activity regularly. The conducted studies show that contemporary Polish young mothers living in rural areas reasonably manage their time. That is why less often they declare the lack of free time than ten years ago [42].

The hypothesis that the lack of financial resources is a barrier for young mothers from rural areas to perform physical activity has been confirmed. The low economic status of young families is often the cause of young mothers’ reluctance and the lack of pleasure and motivation to participate in physical activities [43, 44]. On the other hand, the hypothesis that the lack of access to sports and recreation facilities is a reason for non-participation in physical activity has been not proven. It is worth emphasizing that the previous studies [25, 45] indicated that environmental factors and the lack of access to sports and recreation infrastructure exerted a negative impact on the level of participation in physical activity of women from rural areas. The conducted studies showed that young mothers living in rural areas, to a significantly greater extent than women from urban areas, felt the lack of support from their families. Thus, this part of research hypothesis has been proven. The conducted studies confirmed that women from rural areas wanted to strengthen their position in the family and their self-esteem [46, 47]. This also applied, with even greater intensity, to women from urban areas. It can be stated that women, in general, feel unappreciated by their families [48, 49]. The above mentioned issues seem to be even more important than more traditional problems as weight loss, improving the beauty or creating healthy lifestyle by participating in physical activity. The answers given by the respondents show that the motherhood significantly decreases participation in physical activity, what is confirmed in studies carried by other authors [50–52].

## Conclusions

The study showed that the level of physical activity of young Polish mothers from rural and urban areas was at the same level. However, the differences appeared when choosing the type of physical activity. The priority for young mothers and their families is the growth of their financial resources, thus the raise of their material status. Young mothers, to some extent, should have less daily duties in order to be able to participate in physical activity. This will help them reduce stressful situations and will have a positive effect on restoring a sense of control over their own health and well-being. Young mothers, especially from urban areas, should have more support from their families, especially from their husbands in raising children. This may eliminate stereotypes of young mothers who do not have any free time and strengthen their position in the family and society. The elimination of barriers associated with infrastructure of rural areas such as building bicycle and pedestrian paths, will help young people, including young mothers, spend their free time actively. This social support with the support of a spouse and family are factors which should have a positive influence on active behavior of young mothers.

## Supplementary information

**Additional file 1.**

## Data Availability

The data supporting our findings are found at, kept in confidentiality and stored at the corresponding author both in hard and soft copies. If someone wants our data, we are voluntary to share it and the corresponding author should be contacted through the email address on the cover page.

## Abbreviations

IPAQ: International Physical Activity Questionnaire
MET-min./week: The 1 MET metabolic equivalent corresponds to O_2_ consumption at rest and equals 3.5 ml O_2_ / kg body weight per minute
BMI: *Body Mass Index*
