# Supplementary material for: Identification of motives and barriers to physical activity of polish young mothers
Source: BMC Womens Health. 2020 Sep 11;20:197. doi: 10.1186/s12905-020-01061-y (PMC7488306; doi:10.1186/s12905-020-01061-y)
Supplement: Supplementary file 1 — Additional file 1. [file 12905_2020_1061_MOESM1_ESM.docx]

***Appendix No. 1***

***Survey questionnaire on physical activity of women after giving birth***

1. **How many hours of free time do you have on weekdays?** (on average on each of these days)? (free time is understood as time after completing school duties, work and home duties)

| **Answer** | | select - X |
| --- | --- | --- |
| 1. | Below 2 hours, |  |
| 2. | From 2 to5 hours, |  |
| 3. | Above 5 hours. |  |

1. **How many hours of free time do you have on weekends?** (on average on each of these days)?

| **Answer** | | select - X |
| --- | --- | --- |
| 1. | Below 2 hours, |  |
| 2. | From 2 to5 hours, |  |
| 3. | Above 5 hours. |  |

1. **Please specify your free time budget.**

| **Answer** | | select - X |
| --- | --- | --- |
| 1. | Ii is more than enough, |  |
| 2. | It is enough, |  |
| 3. | It could be more, |  |
| 4. | It is not enough, |  |
| 5. | No free time. |  |

1. **What is the barrier that hinder you from spending free time actively? (Select the correct numeric responde to each questions. Please note that 1 is less likely and 5 most likely)**

| **Answer** | | 1 | 2 | 3 | 4 | 5 |
| --- | --- | --- | --- | --- | --- | --- |
| 1. | No financial resources, |  |  |  |  |  |
| 2. | No offers of sport and recreational centers, |  |  |  |  |  |
| 3. | No interests in offers of sport and recreational centers, |  |  |  |  |  |
| 4. | Timidity |  |  |  |  |  |
| 5. | No family support, |  |  |  |  |  |
| 6. | The lack of free time, |  |  |  |  |  |
| 7. | No access to sport facilities, |  |  |  |  |  |
| 8. | Health problems. |  |  |  |  |  |

1. **What is your motivation to spend your free time activity? (Select the correct numeric responde to each questions. Please note that 1 is less likely and 5 most likely)**

| **Answer** | | 1 | 2 | 3 | 4 | 5 |
| --- | --- | --- | --- | --- | --- | --- |
| 1. | Promoting healthy lifestyle, |  |  |  |  |  |
| 2. | Improving health condition, |  |  |  |  |  |
| 3. | Improving the beauty, |  |  |  |  |  |
| 4. | Improving well-being, |  |  |  |  |  |
| 5. | Strengthening immunity, |  |  |  |  |  |
| 6. | Improving self-confidence, |  |  |  |  |  |
| 7. | Body hardening, |  |  |  |  |  |
| 8. | Relieving the pain, |  |  |  |  |  |
| 9. | Strengthening family position, |  |  |  |  |  |
| 10. | Reducing stress, |  |  |  |  |  |
| 11. | Dieting. |  |  |  |  |  |
